# Multi-omics integration analysis of leaves reveals the adaptive survival mechanism of *Carpinus oblongifolia*, an endemic tree species in subtropical forests of eastern China

**DOI:** 10.3389/fpls.2026.1920434

**Published:** 2026-09-03

**Authors:** Sumei Li, Shuan Wang, Peng Wang, Rutong Yang, Naiwei Li

**Affiliations:** Jiangsu Key Laboratory for Conservation and Utilization of Plant Resources, Institute of Botany, Jiangsu Province and Chinese Academy of Sciences, Nanjing Botanical Garden Memorial Sun Yat-Sen, Nanjing, Jiangsu, China

**Keywords:** *Carpinus oblongifolia*, endemic species, leaf thickness, metabolomics, P limitation, survival strategy, transcriptomics

## Abstract

**Introduction:**

The soils of subtropical forests are generally limited by P availability, and over time the P limitation experienced by the tree species becomes increasingly severe. The plasticity of leaf traits and the reprogramming of metabolites are strategies for plants to adapt to environmental stress, including P limitation. However, research that link the plasticity of leaf traits, gene regulation, and metabolism to reveal the adaptive survival mechanisms to P limitation is still relatively scarce.

**Methods:**

The structural and chemical traits, transcriptomics, and metabolomics of the leaves of an endemic species, *Carpinus oblongifolia* (Betulaceae), naturally growing in a P-limited subtropical forest of eastern China, were investigated.

**Results and Discussion:**

P limitation and pH was the predominant soil factor driving the variation in leaf traits of *C. oblongifolia*. The plasticity of leaf thickness (LT) was the core ecological strategy for this species to adapt to P-limited habitats and leaf thickening was closely related to the accumulation of secondary metabolites. Further, through integrated analysis of transcriptomics and metabolomics, the regulatory network involving four hub genes and five metabolites was constructed to mediate leaf thickening. The coordinated adaptation in leaf structure and metabolism achieves a balance between resource utilization efficiency and stress tolerance, which were the adaptive survival mechanisms of *C. oblongifolia* in P-limited subtropical forest ecosystems. This study has for the first time revealed the adaptive survival mechanisms of *C. oblongifolia*, providing a new perspective on ecological restoration, conservation planning, and genetic resource management of a species with small population under global climate change.

## Introduction

Soil nutrients are fundamental in shaping ecosystem productivity and response to global change. Plants response to soil nutrient changes generally by shifting functional traits along the continuum of the leaf economic spectrum from resource rapid acquisition and long-term conservation (Ordoñez et al., 2009). Structural traits, such as specific leaf weight (SLW) or specific leaf area (SLA), and leaf dry matter content (LDMC), along with chemical traits like leaf nitrogen (LN) and phosphorus (LP) concentrations, are the key features of leaf trait economic spectrum that modulate the performance of plants via influencing their growth, survival, and reproduction (Wright et al., 2004). The relationship between soil nutrients and leaf traits indicates a trade-off between growth rate and nutrient conservation. The species with conservative leaves (i.e. traits such as high SLW and LDMC and thick leaves) are often associated with soils of low-nutrient availability (Ordoñez et al., 2009; Jager et al., 2015). Leaf thickness (LT), an indispensable aspect of leaf three-dimensional morphological structure, strongly influences plant fitness via modulation of major physiological processes. Due to its physiological relevance, LT plasticity is a promising adaptation to environmental change (Aneja et al., 2025).

Soil P constraint has a strong effect on leaf traits at global scale (Du et al., 2020). Especially in subtropical forests, soil P plays a predominant role in driving the variation in plant functional traits (Cui et al., 2022). The P limitation of subtropical forests is projected to become more severe with N deposition and climate warming (Yu et al., 2018; Wang et al., 2019). The P limitation experienced by the tree species has gradually intensified over time in the subtropical region in eastern China (Li et al., 2025). Therefore, clarifying the relationship between soil nutrients and leaf traits is crucial issue in predicting the adaptive evolution of tree species and plant community succession in the context of global climate change.

Genetics and environmental factors serve as two primary determinants that influence the variation in leaf traits. Environmental factors usually induce the variation in leaf traits by affecting the accumulation of metabolites with important biological functions (Zhou et al., 2022). Under different environmental conditions, the types of metabolites produced and the genes activated by plants vary significantly. The content and activity of metabolites depend on the soil in which the plant grows, thereby influencing the survival strategies of plants by regulating resource allocation—prioritizing the synthesis of either defense substances or sugars necessary for growth (Shen et al., 2023). Therefore, the metabolome serves as a biochemical bridge connecting genetic factors with environment-driven trait variation. Secondary metabolites are not essential for the survival of the plant, but they play an important role in adaptability to the environment. For instance, flavonoids, a significant class of secondary metabolites widely present in plants, possess various ecological functions such as antioxidant defense and signal regulation, helping plants transform external stimuli such as UV radiation, drought, high temperature, and salt stress, into adaptive changes at the metabolic and physiological levels (Ren et al., 2026). However, it is not yet clear whether there were any specific secondary metabolites involved in the adaptive shift of leaf traits under P limitation, as well as the genetic regulatory mechanisms involved therein.

*Carpinus oblongifolia* (Hu) Hu & W. C. Cheng (Betulaceae), a monoecious deciduous tree species, is endemic to the Baohua Mountain National Nature Reserve, a subtropical forest of eastern China. Based on a recent field investigation by Li et al. (2020), *C. oblongifolia* is one of several associated species in the secondary forest community. High stand density and strong inter-specific competition have severely limited the number of *C. oblongifolia* trees. Beyond this, human activities associated with the development of tourism have further fragmented the distribution of natural population and created more complex habitats. As a result, the wild population, currently consisting of approximately 130 trees, is fragmented on northeast-facing hillsides with extremely limited natural renewal capacity and genetic diversity (Li et al., 2022). Due to its limited population size, habitat fragmentation, and poor natural regeneration capacity, this species has been listed as a locally endemic species and a “critically endangered” plant by the Chinese Biodiversity Red List – Higher Plants, jointly compiled by the Ministry of Environmental Protection and the Chinese Academy of Sciences (http://www.iplant.cn/rep/prot/Carpinus%20oblongifolia).

The Baohua Mountain ecosystem, like other subtropical forests in China, suffers from soil P limitation (Wang et al., 2019). The soil available phosphorus (SAP; 1.04–6.07 mg kg^−1^) of *C. oblongifolia* habitats was much lower than the average value of 12.40 mg kg^−1^ in global surface soil (Luo et al., 2025). Nutrient stress may be one of the reasons restricting the development of the *C. oblongifolia* population. However, previous research mainly focused on the investigation of the current status of wild population, the effect of soil nutrients in the habitat on the structural and chemical traits of *C. oblongifolia* leaf, as well as involving the related genes and metabolites, remain elusive. We hypothesized that: (1) P limitation was the predominant soil factor driving the leaf traits variation of *C. oblongifolia*; (2) LT plasticity, an important adaptation under changing climate, was also a core adaptive trait for *C. oblongifolia*; and (3) secondary metabolites served as the biochemical basis for the LT plasticity under P limitation. The objective of our research was to elucidate the adaptive survival mechanism of *C. oblongifolia* to the local environment, offering a scientific basis for formulating conservation plans for sustainable development of the small population in light of global climate change.

## Materials and methods

### Study area and plant materials

This study was conducted at the Baohua Mountain (31°37'–32°19'N, 118°58'–119°58'E), a northern subtropical forest national nature reserve located near the city of Zhenjiang, Jiangsu Province, China. The Baohua Mountain has a typical vegetation of mixed deciduous and evergreen broadleaved forests, with the highest peak at 396 m above sea level (An et al., 2001). The area is characterized by a well-preserved ecosystem with a vegetation coverage rate as high as 92% and a rich variety of plant species, preserving several wild rare plant resources, such as *Yulania zenii*, *Changnienia amoena*, and *Sinojackia xylocarpa*. The parent soil of the Baohua Mountain consists of limestone, sandstone, and shale, which has developed into zonal yellow-brown soil with moderate degree of weathering. The climate is mild and humid, and belongs to the humid monsoon climate zone in the northern-subtropics. The annual average precipitation is 1,088 mm, with rainfall mainly concentrated in July and August, which includes up to about half of the total annual precipitation. The annual average temperature is 15.4 °C.

In a previous study, the wild *C. oblongifolia* population was artificially divided into four subpopulations based on their habitats for genetic diversity analysis (Li et al., 2022). The basic information such as soil nutrient contents of the four habitats is shown in **Table 1**. The present study further analyzed the adaptive survival mechanism characterized by the leaf traits associated with soil nutrients based on these four subpopulations. To better represent the consistency of the research subjects, three trees with a diameter of around 10 cm at breast height were selected from each subpopulation as the objects of study, and at least 10 healthy, fully expanded and sun-exposed leaves at a height of 3 m were collected from four directions (east, west, north, and south) in the middle of the crown of each tree by an averruncator. All the collected leaves were placed in plastic self-sealing bags within an ice cooler to prevent the leaves from losing water; the samples were then transferred to the laboratory for subsequent analysis.

**Table 1:** The basic information of four *Carpinus oblongifolia* sampling habitats.

Factors	H1	H2	H3	H4
Latitude and longitude	119°05′04″E, 32°07′57″N	119°05′13″E, 32°07′59″N	119°05′03″E, 32°07′59″N	119°05′25″E, 32°07′49″N
Specific position	In the forest	In the forest	Roadside	In the forest
Crown density (%)	98.13	79.34	72.46	86.27
Dominant species	*Gleditsia japonica*	*Quercus acutissima*	*Liquidambar formosana*	*Quercus acutissima*
Gravel content of soil (%)	25.34	25.34	21.68	30.59
pH (H_2_O)	7.41 ± 0.06 b	6.75 ± 0.36 c	7.94 ± 0.07 a	7.68 ± 0.12 a
SOC (g·kg^−1^)	25.82 ± 0.98 b	15.53 ± 0.24 d	22.58 ± 0.96 c	28.01 ± 0.68 a
STN (g·kg^−1^)	2.28 ± 0.04 a	1.65 ± 0.06 c	1.95 ± 0.02 b	2.44 ± 0.15 a
STP (g·kg^−1^)	0.52 ± 0.02 b	0.63 ± 0.01 a	0.30 ± 0.05 c	0.57 ± 0.03 ab
STK (g·kg^−1^)	14.33 ± 0.44 b	8.46 ± 0.21 c	14.92 ± 0.61 b	18.03 ± 0.21 a
SAN (mg·kg^−1^)	233.61 ± 4.06 a	170.36 ± 13.94 bc	159.38 ± 2.77 c	182.90 ± 6.65 b
SAP (mg·kg^−1^)	4.31 ± 0.25 b	6.07 ± 0.48 a	1.04 ± 0.03 c	5.95 ± 0.36 a
SAK (mg·kg^−1^)	103.70 ± 19.02 b	115.63 ± 1.93 b	182.10 ± 4.88 a	72.43 ± 4.99 c

Different lowercase letters indicate significant differences among the habitats at *P* < 0.01 level. SOC, soil organic carbon; STN, soil total nitrogen; STP, soil total phosphorus; STK, soil total potassium; SAN, soil available nitrogen; SAP, soil available phosphorus; SAK, soil available potassium.

### Leaf trait measurement

The leaf area (LA, cm^2^), SLA (cm^2^ g^–1^), LDMC (g g^–1^), LN (g kg^–1^), and LP (g kg^–1^) were measured using the protocol of Cornelissen et al. (2003). The leaf thickness (LT, µm), the palisade tissue thickness (PTT, µm), and the spongy tissue thickness (STT, µm) were measured using the paraffin section method. Then the stoichiometric values of PTT: STT (PSR) and LN: LP (LNP) were calculated individually. Finally, a plasticity index was calculated according to the method of Valladares et al. (2006), where the plasticity index of a trait= [(max – min)/max]; this index is a dimensionless parameter, with a greater value indicating stronger plasticity.

### RNA sequencing and WGCNA

RNA extraction, library construction, and RNA sequencing were performed by Genedenovo Biotechnology Co. Ltd. (Guangzhou, Guangdong, China) according to standardized protocols. The Unigene sequences were compared with the following protein databases: the Non-Redundant protein sequence (NR), the Kyoto Encyclopedia of Genes and Genomes (KEGG), the Eukaryotic Orthologous Groups of Proteins, and Swiss-Prot using the BlastX bioinformatics tool to obtain functional annotations. Following the exclusion of genes with low expression levels, genes with greater than 2.5 fragments per kilobase of sequence per million mapped reads were used to conduct WGCNA using Omicsmart, a real-time interactive online platform for data analysis (http://www.omicsmart.com). In WGCNA, the soft threshold was set to 12 based on the signed r^2^ = 0.8 in the scale-free topology fit plot (**Supplementary Figure 1**), mergeCutHeight to 0.2, minModuleSize to 50, and module similarity to 0.85 to construct a gene-trait network based on the correlations between leaf traits and the module eigengenes. To identify the hub genes within the modules, the module membership (MM) for genes and the module eigengenes, and the gene significance (GS) for genes and leaf traits were calculated based on the Pearson correlation. The topological overlap value between genes was calculated based on an adjacency matrix to reflect the gene connectivity in each module. The Cytoscape (v.3.10.1) platform was then used to visualize the gene networks within each module and present the biological interactions of the genes.

### Metabolomic analysis

Metabolomic profiles of 12 leaf samples identical to those of the transcriptomes were generated using a non-targeted metabolomics method. The analytical protocol comprised three sequential phases: compound extraction, chromatographic separation coupled with HPLC-MS/MS platforms, and bioinformatics processing for compound annotation, all of which were conducted under standardized protocols at Genedenovo Biotechnology Co. Ltd. In brief, the fresh leaf sample was extracted using extraction solution (methanol:acetonitrile:water = 2:2:1, v/v/v). Then, the supernatant was analyzed using a Vanquish UHPLC System (ThermoFisher, Waltham, MA, USA) coupled to an Orbitrap Exploris 120 mass spectrometer (ThermoFisher). Mass data acquisition was performed in both positive [electrospray ionization-positive (ESI^+^)] and negative (ESI^–^) modes using the following parameters: ion spray voltage of 3.8 kV in ESI^+^ and −3.4 kV in ESI^–^, sheath gas flow rate as 50 Arb, Aux gas flow rate as 15 Arb, capillary temperature at 320 °C, sweep gas as 1 Arb, vaporizer temperature at 350 °C, full MS resolution as 60,000, MS/MS resolution as 15,000, collision energy as SNCE 20/30/40, and scan range as 70–1,200 Da. The raw data were preprocessed through a systematic pipeline and converted to MzXML files using MSConvert in ProteoWizard software (v.3.0.10200). The converted raw data were further processed using freely available eXtensible Computational Mass Spectrometry software (XCMS software; https://xcmsonline.scripps.edu/landing_page.php?pgcontent=mainPage) and output as a retention time m/z dataset.

### Construction of gene-metabolite regulatory networks

Pearson correlation coefficients (PCC) were used to integrate the metabolomic and transcriptomic data. For this, PCA and orthogonal partial least squares discriminant supervised multivariate pattern recognition models were systematically applied. Differentially expressed metabolites (DEMs) among habitats were screened out based on |log_2_(fold change)| ≥ 1, variable importance in projection ≥ 1, and *P* < 0.05. Gene-metabolite co-expression was analyzed between all expressed genes and all identified DEMs, and the regulatory networks were generated between the hub genes in WGCNA and their target metabolites.

### Statistical analysis

A one-way analysis of variance and a least significant difference multiple comparison test were conducted using R software (v. 4.0.2) to ascertain the differences in each trait among habitats where *P* < 0.05 was considered statistically significant. The correlation heatmap, PCA, and redundancy analysis (RDA) were performed on the Omicsmart data analysis platform (http://www.omicsmart.com). The genetic diversity parameters including expected alleles (Ae), observed heterozygosity (Ho), expected heterozygosity (He), genetic diversity index (Ne), Shannon’s diversity index (I), polymorphism information content (PIC) and inbreeding coefficient (Fis) used in RDA were directly extracted from our previous research based on the single nucleotide polymorphism markers of 10 samples for each subpopulation in a specific length amplified fragment sequencing library (Li et al., 2022). To be consistent with the sample size of this study, the genetic diversity parameters of each subpopulation were replicated three times (**Supplementary Table 1**). PCC analysis was used to calculate correlations between variables. Variance partitioning analysis that quantified the proportions of factors on the variation in traits was conducted using R software.

## Results

### Leaf trait variation across habitats

**Figure 1:**
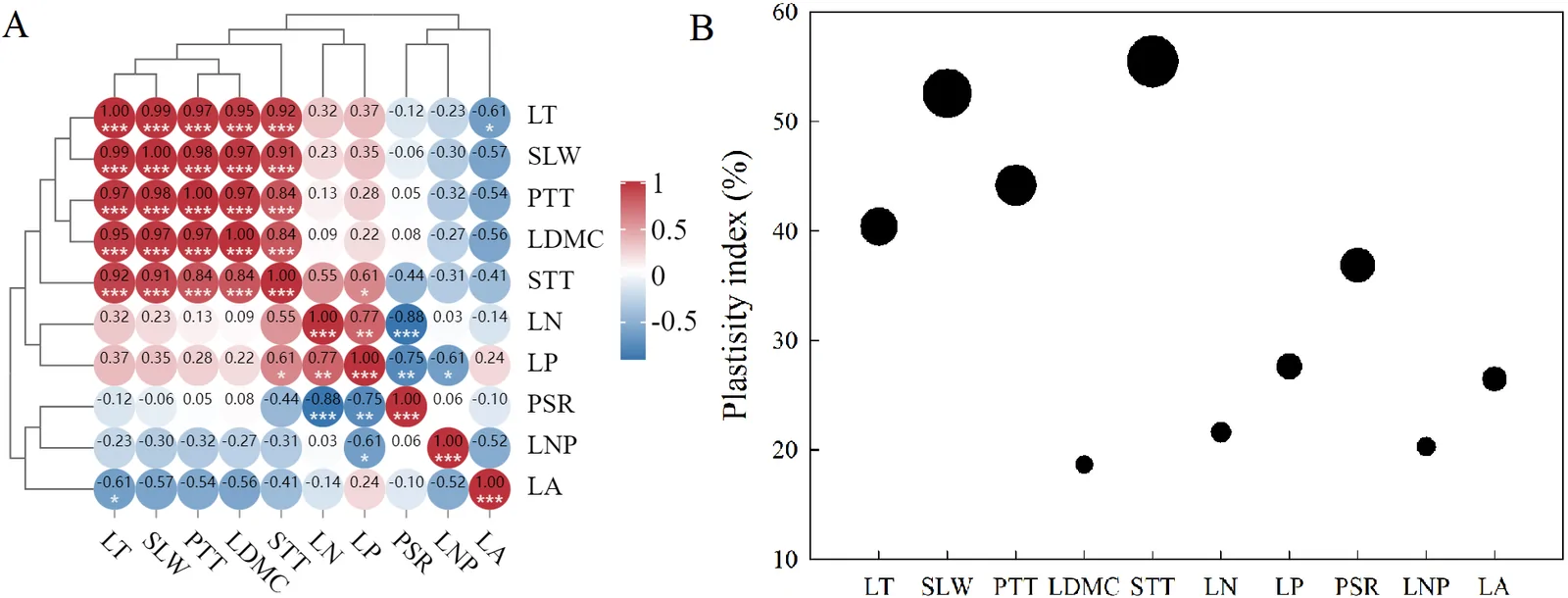
Correlation heatmap **(A)** and plasticity index **(B)** of 10 leaf traits. Red and blue in **(A)** indicate a positive and negative correlations between variables; the numbers and asterisks at the intersection of the two variables represent the correlation coefficients and the level of statistical significance (***P < 0.001, **P < 0.01, *P < 0.05), which is the same as in the following Figures. The size of the dots in **(B)** is directly proportional to the plasticity index.

All leaf traits studied exhibited significant differences to varying degrees among the four habitats (**Supplementary Figure 2**). Specifically, the variation trend of LT, PTT, LDMC and SLW was similar among habitats with H3 > H4 > H1 > H2. As a result, LT, SLW, PTT and LDMC clustered into a covariance trait combination, exhibiting an extremely significant positive correlation among them (PCC > 0.90; **Figure 1A**). Likewise, the cluster of LN and LP showed an obvious negative correlation with PSR. Furthermore, a plasticity index was used to quantify the range of variation in the leaf traits among four habitats. Based on plasticity index, the plasticity of each trait was arranged in sequence as STT > SLW > PTT > LT > PSR > LP > LA > LN > LNP > LDMC, with the maximum value of 55.48% and the minimum value of 18.63% (**Figure 1B**).

### Driving factors of leaf trait variation

**Figure 2:**
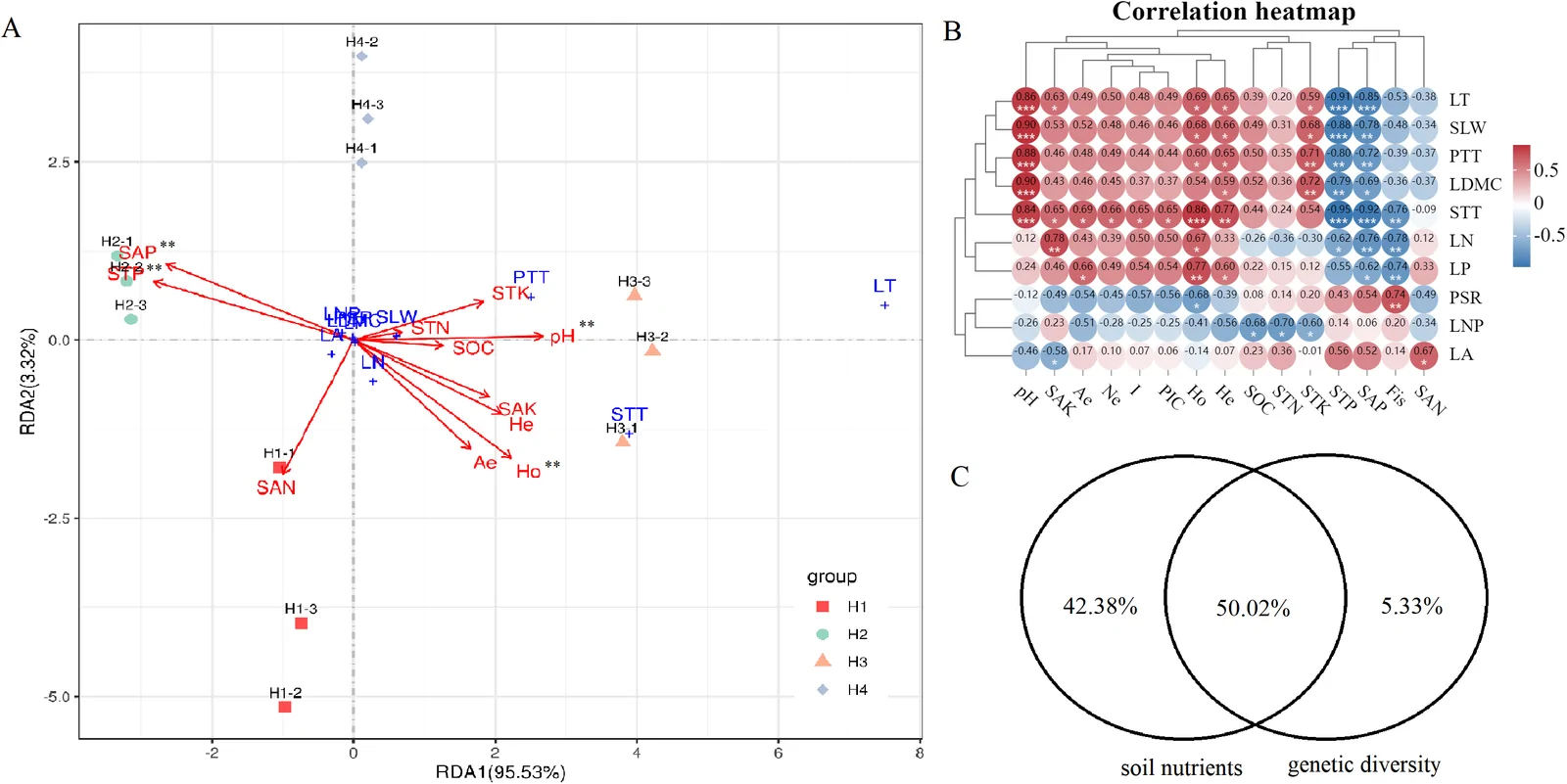
The driving factors of the variation in leaf traits by RDA ranking **(A)**, Pearson correlation heatmap **(B)**, and variance decomposition analysis **(C)** in terms of soil nutrients and genetic diversity parameters. SOC, soil organic carbon; STN, soil total nitrogen; STP, soil total phosphorus; STK, soil total potassium; SAN, soil available nitrogen; SAP, soil available phosphorus; SAK, soil available potassium; He, expected heterozygosity; Ho, observed heterozygosity; Ae, expected alleles. ***P < 0.001,**P < 0.01, * P < 0.05

The genetic diversity parameters extracted from our previous report (**Supplementary Table 1**; Li et al., 2022) combined with soil nutrients of habitats were used to analyze the main driving factors of the leaf trait variation. The RDA indicated that genetic and soil variables accounted for 95.53% variation in leaf traits on the first axis (**Figure 2A**). Among them, the effects of pH, soil total phosphorus (STP), SAP and Ho were the most significant. To explore the effect of different habitats on the robustness of the results, we performed a subset analysis to examine whether the correlations changed when specific site was excluded. The results indicated that pH, SAP, and STP maintained a high robustness, while SAN, STN, and STK exhibited deviations in different subsets analysis (**Supplementary Figure 3**). Furthermore, the correlation analyses showed that the traits of LT, SLW, PTT, LDMC and STT were strongly correlated with pH, STP, and SAP, as well as no significant correlation with STN and SAN (**Figure 2B**). These analysis confirmed a significant correlation between soil P and pH with leaf traits. The variance decomposition analysis indicated that the contribution rate of soil nutrients alone was 42.38%, approximately eight times that of the genetic diversity alone (5.33%), while the combined effects of these two accounted for 50.02% (**Figure 2C**).

### Construction and analysis of the gene-trait network

**Figure 3:**
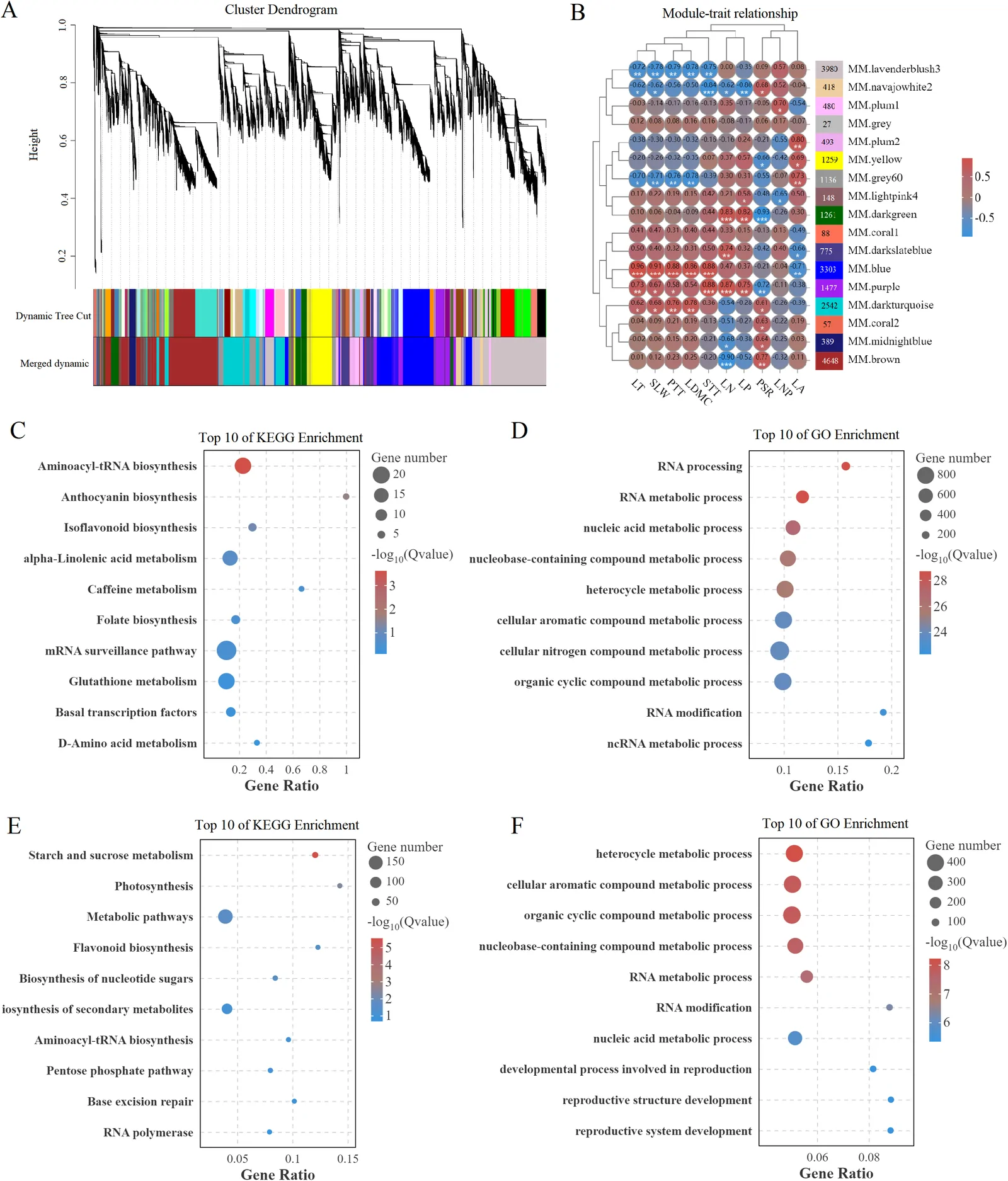
Cluster dendrogram of gene-trait co-expression modules identified by WGCNA **(A)**, module-trait correlations heatmap **(B)**, KEGG enrichment of the MM.blue module genes **(C)**, GO enrichment analysis of the MM.blue module **(D)**, KEGG enrichment of the MM.purple module genes **(E)**, and GO enrichment analysis of the MM.purple module **(F)**. The merged dynamic tree in **(A)** constitutes 17 modules labeled with different colors for the subsequent analysis. The numbers in the module color box in **(B)** indicate the number of genes it contains. ***P < 0.001,**P < 0.01, * P < 0.0.

A total of 42,383, 39,630, 21,317 and 26,401 annotated unigenes were obtained respectively from the NR, KEGG, the Eukaryotic Orthologous Groups of Proteins, and Swiss-Prot databases after performing RNA sequencing (**Supplementary Table 2**). The transcriptome data of three samples from each habitat clustered into a separate group, with a considerable number of differentially expressed genes between each other (**Supplementary Figure 4**). Finally, 22,481 unigenes were obtained after filtering out low-quality genes and were used to construct a gene-trait co-expression network module (**Supplementary Table 3**). Seventeen co-expression modules were constructed based on the module eigengene (**Figure 3A**). The module-trait relationship indicated that the MM.blue module presented a highly significant positive correlation to LT, SLW, PTT, LDMC and STT, with the correlation coefficients of 0.96, 0.91, 0.88, 0.86 and 0.86 respectively (*P* < 0.001), as well as the MM.purple module presented a highly significant positive correlation to STT and LN and the correlation coefficients were 0.88 and 0.87 respectively (*P* < 0.001; **Figure 3B**). Through enrichment analysis, the genes within MM.blue module were mainly enriched in the pathways of aminoacyl-tRNA biosynthesis and anthocyanin biosynthesis (**Figure 3C**), involving in RNA processing and RNA metabolic process (**Figure 3D**), while the genes within MM.purple module were mainly enriched in the pathway of starch and sucrose metabolism (**Figure 3E**), involving in various metabolic process (**Figure 3F**).

The MM.blue module contained 3,303 genes (**Supplementary Table 4**) and the GS for LT exhibited the strongest linear correlation with MM (r = 0.80), while the GS-MM correlations for STT, SLW, PTT, and LDMC were 0.67, 0.65, 0.56, and 0.55 respectively (**Supplementary Figure 5**). Among the genes with the top 10% connectivity, based on |GS| > 0.90 (*P* < 0.0001), 118 genes were found to have a high GS for LT, while STT and SLW had only 32 and 45 genes respectively. Moreover, all the genes for STT and SLW were included within the genes for LT (**Supplementary Table 5**). These results indicated that the genes in MM.blue module have the strongest explanatory power for LT. The MM.purple module contained 1,477 genes (**Supplementary Table 6**), and the GS-MM correlations for STT and LN were 0.69 and 0.72 respectively (**Supplementary Figure 6**). Among the genes with the top 10% connectivity, 37 and 22 genes were found to have a high GS for STT and LN respectively based on |GS| > 0.90 (*P* < 0.0001), but these genes did not overlap each other (**Supplementary Table 7**), reflecting that the GS value in the MM.purple module exhibited specificity for different traits.

**Figure 4:**
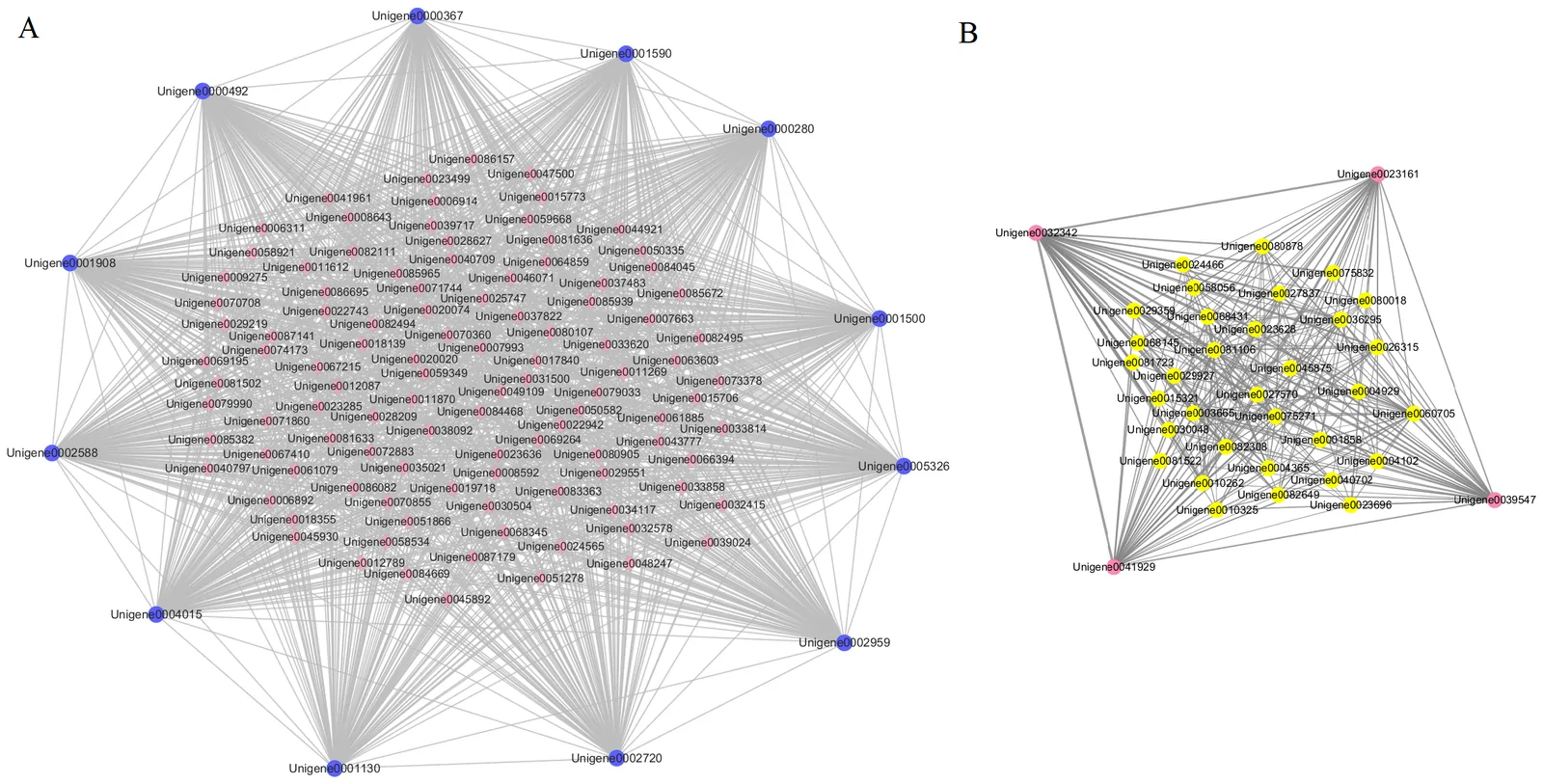
Analysis of the interactions between gene networks within the MM.blue module **(A)** and the MM.purple module **(B)**. The larger nodes are highly connected within the module and are selected as the hub genes.

The network visualization map of genes with the edge weight > 0.30 in the module was drawn via Cytoscape software. Twelve genes with a degree > 100 were identified as the hub genes of the MM.blue module (**Figure 4A**) and four genes with a degree > 30 were identified as the hub genes of the MM.purple module (**Figure 4B**). Among the 12 hub genes in the MM.blue module, Unigene0000280 and Unigene0002959 are two genes with unknown functions, while four genes (Unigene0000367, Unigene0000492, Unigene0001130 and Unigene0001590) are involved in the regulation of gene expression, as well as two genes (Unigene0001908 and Unigene0002588) are involved in metabolic processes (**Table 2**). In the MM.purple module, Unigene0023161 is an unknown function gene, while Unigene0032342 is a gene involved in metabolic process, Unigene0039547 is a gene involved in response to stress, and Unigene0041929 is involved in the regulation of gene expression. Notably, the four hub genes of the MM.purple were all |GS| > 0.90 for STT, indicating that the core architecture of its co-expression network was highly biased toward STT.

**Table 2:** The detailed information of the hub genes within the MM.blue module and the MM.purple module.

Module	Gene ID	Description	Biological function	Degree
MM.blue				
	Unigene0000367	GTP-binding protein	Regulation of transcription	116
	**Unigene0000492**	**large ribosomal subunit protein eL15z-like**	**Translation**	**114**
	Unigene0001130	uncharacterized protein LOC132185257	DNA-mediated transposition	114
	**Unigene0001590**	**Eukaryotic translation initiation factor 3 subunit M**	**Cytoplasmic translation**	**112**
	**Unigene0001908**	**monooxygenase 2-like isoform X2**	**Metabolic process**	**111**
	Unigene0002588	probable inactive purple acid phosphatase 27	Phosphorus metabolic process	110
	Unigene0001500	Dipeptide transport ATP-binding protein dppF	ATP-binding	113
	Unigene0002720	monothiol glutaredoxin-S15	Cell redox homeostasis	109
	Unigene0005326	perakine reductase-like	Potassium ion transport	105
	Unigene0000280	hypothetical protein FH972_015772	–	115
	Unigene0002959	hypothetical protein FH972_012221	–	108
	Unigene0004015	containing deubiquitinating enzyme 5-like	–	107
MM.purple				
	Unigene0023161	UV-B-induced protein At3g17800	–	35
	**Unigene0032342**	**cytochrome c-type biogenesis ccda-like chloroplastic protein**	**Metabolic process**	**32**
	Unigene0039547	probable zinc metalloprotease EGY1, chloroplastic	Response to stress	36
	Unigene0041929	tryptophan--tRNA ligase	Translation	32

the bolded genes are those that are involved in the gene-metabolite regulatory network.

### Analysis of gene-metabolite regulatory networks

**Figure 5:**
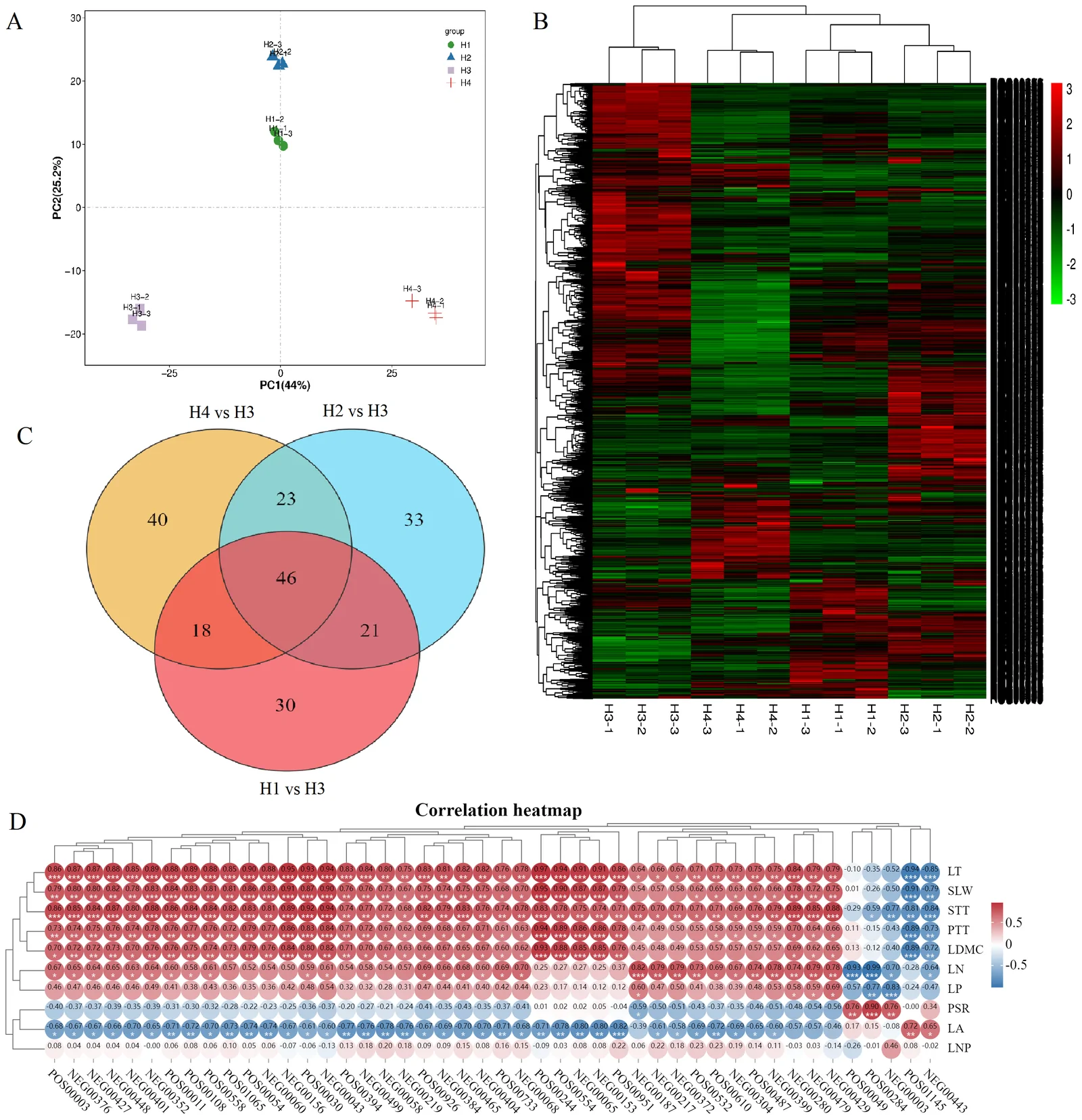
PCA **(A)** and cluster analysis **(B)** of samples based on metabolomic data, multiple comparative analysis of the DEMs between H3 with the other three habitats **(C)**, and the correlation heatmap between 46 DEMs and 10 leaf traits **(D)**. ***P < 0.001,**P < 0.01, * P < 0.05.

More than 1,700 metabolites were identified in positive and negative ion modes (**Supplementary Table 8**), which showed strong correlations among the biological replicates of each habitat through PCA and cluster analysis (**Figures 5A, B**). To identify a general trend in the metabolome associated with soil nutrients, H3 with the lowest STP and SAP was set as a control for comparison analysis. Within the thresholds of variable importance in projection ≥ 1, |log_2_(fold change)| ≥ 1 and *P* < 0.05, 115, 123, and 127 DEMs were identified from H1 vs H3, H2 vs H3, and H4 vs H3, respectively. Multiple comparative analysis showed 46 metabolites were the common DEMs between H3 and the other three habitats (**Figure 5C**), including 14 flavonoids (30.43%), eight alkaloids (17.39%), four fatty acids (8.70%), and four terpenoids (8.70%; **Supplementary Table 9**). Further analysis revealed that the abundance of these DEMs exhibited a significant correlation with different leaf traits (**Figure 5D**). Among them, most of the metabolites related to LT were significantly correlated with SLW, STT, PTT, and LDMC.

**Figure 6:**
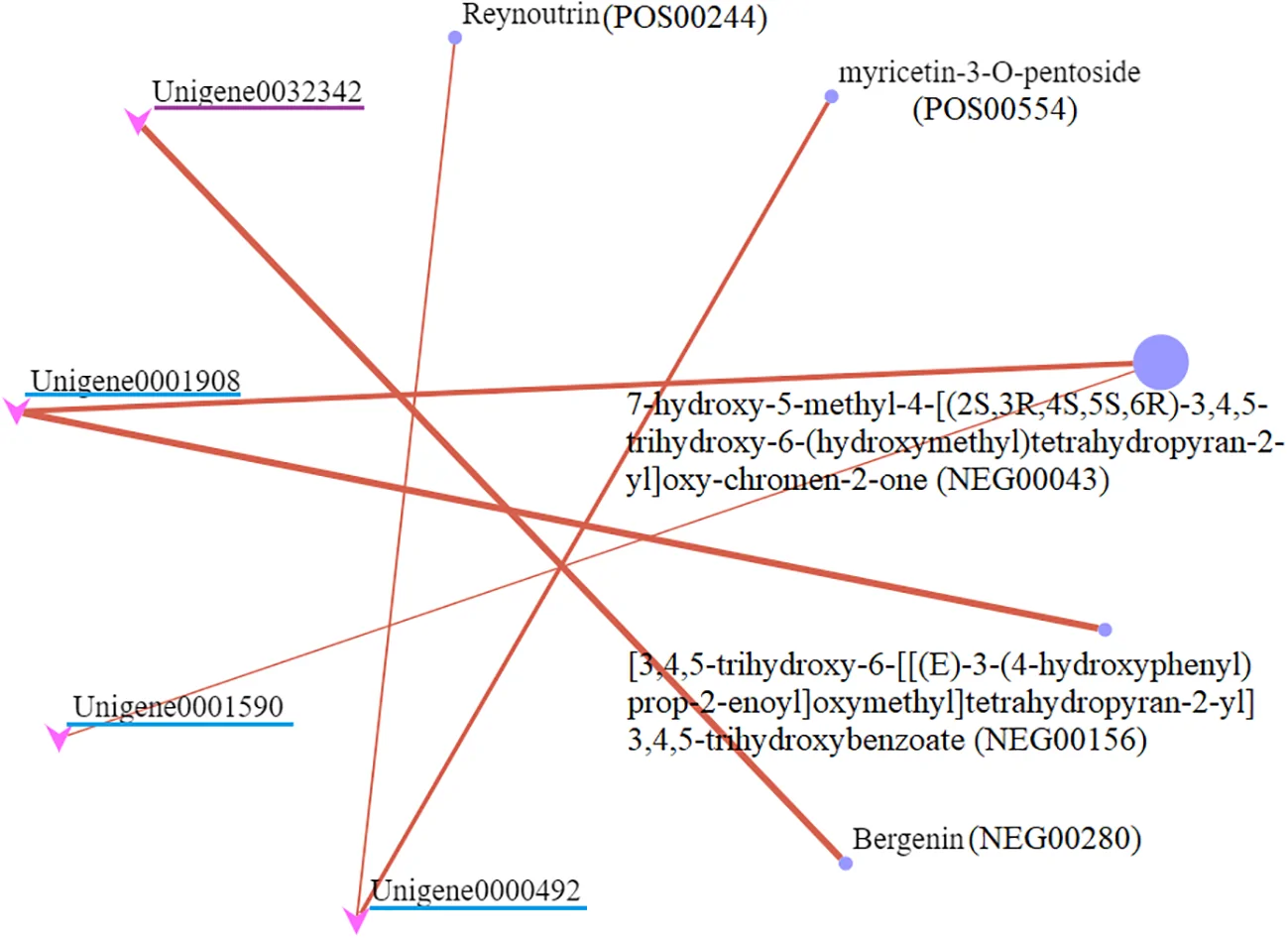
The co-expression network of the four hub genes and five metabolites. The red solid line represents the positive correlation between the genes and metabolites. The genes marked with blue underlines belong to the MM.blue module, and the gene marked with purple underline belongs to the MM.purple module.

To reveal the genetic basis of metabolite accumulation, all expressed genes were used to perform the co-expression analyses with these 46 DEMs, and 461 genes were significantly correlated with 41 DEMs (**Supplementary Table 10**). Among them, 359 (77.87%) and 26 (5.64%) genes were from the MM.blue and the MM.purple module respectively, including four hub genes identified in gene-trait co-expression networks, Unigene0000492, Unigene0001590, Unigene0001908 and Unigene0032342. The four hub genes were directly related to five compounds in the above 41 DEMs, forming a gene-metabolite network (**Figure 6**): Unigene0000492 was positively correlated to reynoutrin (POS00244) and myricetin-3-O-pentoside (POS00554), Unigene0001590 was positively correlated to 7-hydroxy-5-methyl-4-[(2S,3R,4S,5S,6R)-3,4,5-trihydroxy-6-(hydroxymethyl)tetrahydropyran-2-yl]oxy-chromen-2-one (NEG00043), Unigene0001908 was positively correlated to NEG00043 and [3,4,5-trihydroxy-6-[[(E)-3-(4-hydroxyphenyl)prop-2-enoyl]oxymethyl]tetrahydropyran-2-yl] 3,4,5-trihydroxybenzoate (NEG00156), and Unigene0032342 was positively correlated to Bergenin (NEG00280).

## Discussion

### P limitation was the predominant soil factor influencing the leaf trait variation

Our study systematically examined the impacts of soil nutrients and genetic diversity on leaf traits in *C. oblongifolia*: the combined effect of the two was the most significant factor contributing to the variation in leaf traits, which is consistence with the core view of gene-environment interaction in plant ecological adaptation. However, the single explanatory rate of soil nutrients was far greater than that of the genetic diversity, implying that the effective expression of genetic factors was driven by the specific soil conditions because soil P limitation may act as the environmental filter promoting the convergence of traits related to acquisition or conservation of resources (Domínguez et al., 2012). That is, natural selection have had a bigger role than genetic constraints in the evolution of the leaf traits (Donovan et al., 2011). Only those individual plants with the functional traits that promote high fitness and performance can successfully survive and reproduce in the P-limited ecosystem. As a result, the genetic diversity level of the *C. oblongifolia* population is a relatively low (Li et al., 2022), which in turn limits the independent contribution of genetic factors to leaf traits. In this context, in-depth research on the adaptive response to P limitation is crucial for understanding the formation of species distribution boundaries and the evolution of ecological niches.

The ratio of LN to LP (LNP) is a simple indicator for identifying nutrient limitations on plant growth and biomass production (Yan et al., 2017). In this study, LNP of *C. oblongifolia* always remained above 20:1 across four habitats, which is higher than the widely used threshold values of 16:1 or 20:1 (Du et al., 2020), confirming that P limitation is a pervasive soil factor on subtropical tree growth as our system (Cui et al., 2022). The LNP and LP of *C. oblongifolia* was comparable with the average value of the dominant tree species in the eastern subtropical region that are affected by P limitation (Li et al., 2025). Under these conditions, the LT, SLW, and LDMC exhibited a significant increase with the decrease of SAP and STP (**Figure 2B**), suggesting *C. oblongifolia* shifted the leaf traits toward to more conservative values. This P-limiting conservative strategy has been reported on stenotopic native species in tropical forests (Fan et al., 2025), the dominant trees in a subtropical forest (Zhao et al., 2023), a wetland plant community along the Yellow River (Ding et al., 2025), as well as tropical rainforest ecosystems (Chen et al., 2025). These results support the first hypothesis that *C. oblongifolia*, like other species in the ecosystem, experiences substantial changes in leaf traits due to P limitation, adjusting resource utilization strategies.

### LT plasticity was the core adaptive trait

Plants respond to changes in the environment via suits of leaf traits representing trade-offs and co-optimization relationships based on physiological and biochemical requirements for survival (He et al., 2020). First, the presence of covariance correlations among SLW, LT, and LDMC (**Figure 1A**) indicated that these traits were related by causal relationships. SLW, one of the core functional traits for measuring the cost of leaf construction and environmental adaptation strategies, is a function of LDMC and LT. In the present study, the plasticity of LDMC was the lowest, suggesting that *C. oblongifolia* could maintain carbon-nitrogen ratio and metabolic efficiency to ensure basal metabolism and stress resistance in response to environmental changes, which is particularly important for perennial woody plants (Duan et al., 2026). Hence, *C. oblongifolia* tended to modify SLW by altering LT. Species in low-fertility habitats tends to have high LT to adapt to such environments (Zhang et al., 2017; Ding et al., 2025). Moreover, the thickening of leaves is usually found in plants that adapt to hotter, drier, and higher irradiance environments (Poorter et al., 2019). Secondly, LN and LP showed a strong negative correlation with the PSR, rather than individual PTT or STT (**Figure 1A**), implicating that the LT plasticity altered the anatomical attributes of leaves to directly optimize the allocation of resources between photosynthetic growth and defense investment. Thirdly, among the two modules that were most closely related to leaf traits, the MM.blue module had more greater impact on LT (**Figure 3B**; **Supplementary Figures 5**, **6**). Among the top 10% connectivity genes of the MM.blue module, the number of genes with |GS| > 0.90 for LT were more than those for SLW and STT, and the former included all the genes of the latter (**Supplementary Table 5**), indicating that the hub genes of the module are precisely those that are most crucial for LT, while the impact of the module on STT and SLW may be mainly achieved indirectly through the shared genetic basis of LT. Fourthly, the DEMs associated with LT were mainly secondary metabolites (**Figures 5C, D**), indicating a functional correlation between the thickening of the leaves and the accumulation of some defense compounds, as reported in Mediterranean plants (Fernández-Marín et al., 2017). Therefore, LT plasticity achieves a balance between carbon assimilation for growth and secondary metabolism for defense through resource trade-off strategies, and is the core trait for the adaptive growth of *C. oblongifolia* in the local environment.

### The adaptive survival mechanism

Based on the above discussion that soil P limitation drives the plasticity of LT, a combination of transcriptomic analysis and metabolomic analysis provides the molecular foundation for understanding the LT variation of *C. oblongifolia*. Through the implementation of WGCNA and integrative analyses of metabolic and transcriptomic data, a gene-metabolite regulatory network consisting of four genes and five metabolites was constructed, which was directly related to the LT modulation of *C. oblongifolia* response to P-limited environments (**Figure 6**).

As sessile organisms, plants rely on the modulation of diverse metabolites for stress response and defense in varying environments during the growth period. In the gene-metabolite regulatory network, 7-hydroxy-5-methyl-4-[(2S,3R,4S,5S,6R)-3,4,5-trihydroxy-6-(hydroxymethyl)tetrahydropyran-2-yl]oxy-chromen-2-one is a coumarin compound, [3,4,5-trihydroxy-6-[[(E)-3-(4-hydroxyphenyl)prop-2-enoyl]oxymethyl]tetrahydropyran-2-yl] 3,4,5-trihydroxybenzoate is a phenylpropanoid (C6-C3), as well as bergenin, reynoutrin, and myricetin-3-O-pentoside are three flavonol compounds, all belonging to the metabolites of the phenylpropanoid metabolic pathway. At present, there are no reports available regarding the physiological functions of the former two. In this study, their accumulation in leaves of *C. oblongifolia* suggested that they might be regulated as the intermediate products of the phenylpropanoid metabolic pathway. Bergenin is a bioactive compound found in many plant species, exhibiting several biological actions such as antioxidant and anti-inflammatory (Salimo et al., 2023), thereby strengthening the chemical defense system of the *C. oblongifolia* leaves. Reynoutrin, namely quercetin-3-D-xyloside and myricetin-3-O-pentoside are the prevalent flavonol aglycones in the plant kingdom. Flavonols have been shown to regulate plant growth, development, and physiology through two distinct mechanisms: maintenance of reactive oxygen species homeostasis and inhibition of auxin transport (Daryanavard et al., 2023). The concentration of flavonols is closely related to P nutritional status (Jia et al., 2015). P stress results in increased levels of specific quercetin-glycosides in *Arabidopsis thaliana* (Fukushima et al., 2017). It is possible that while eliminating reactive oxygen species induce by P stress, flavonols can be used as indirect growth regulator involving the phytohormone signals such as auxins (Laoué et al., 2022). Quercetin derivatives have been reported to influence the growth and development of *Eucommia ulmoides* leaf shape including leaf length and LA (Meng et al., 2023). Therefore, the synergistic effects of these metabolites regulated by the hub genes enhanced the competitive ability of *C. oblongifolia* in P-limited environments to a certain extent.

Plants regulate phenylpropanoid metabolism through multi-level transcriptional regulation, especially transcription factors such as MYB and WRKY directly target the key genes in biosynthesis (Dong and Lin, 2021). For instance, MYB12 acts as a P starvation response enhancement factor and regulates the expression of flavonol biosynthetic genes, which results in increased flavonols, thereby enhancing tolerance of *Nicotiana tabacum* to low P stress (Song et al., 2020). NtWRKY11b can induce flavonol accumulation through targeting and activating promoter of NtMYB12 (Wang et al., 2021). In this study, Unigene0000492 encoding a large ribosomal protein (RPL) was identified as a hub gene associated with the LT variation. Moreover, it directly regulated the accumulation of reynoutrin and myricetin-3-O-pentoside, suggesting that RPL was involved in the regulatory network that coordinated changes in leaf structure and biochemistry of *C. oblongifolia*. In rice, the expression of RPL genes are highly responsive to stress and signaling molecules such as phytohormone, enabling their encoded proteins to have roles in stress amelioration (Moin et al., 2016). For example, eL15 (OsRPL15) is involved in signal transduction pathways during stress processes (Moin et al., 2021). The regulatory module of PpWRKY31-PpRPL12 in *Pyrus pyrifolia*, ultimately confers resistance to black spot disease by influencing the downstream phenylpropanoid pathways (Cheng et al., 2025), which provides possibility for RPL to participate in the regulation of phenylpropanoid homeostasis. Furthermore, AtRPL12B shows precise adjustments in protein biosynthesis to meet current demands under P or Fe deficiency (Wang et al., 2013), which provides a guarantee for the accurate translation of the key structural genes. This translation reprogramming strategy enables plants to precisely invest limited ribosomes and energy into the optimization of leaf structural traits without having to launch large-scale transcription reprogramming when resources are limited. Altogether, the induced expression of the hub genes regulated the accumulation of phenylpropanoid metabolites, resulting in leaf thickening to strengthen the physical defense, thereby achieving the trade-off for survival. However, this adaptive mechanism is derived from a correlation analysis, and the causal relationship between specific gene and metabolite therein needs to be rigorously verified and clarified at the cellular level in *C. oblongifolia*.

## Conclusion

By analyzing the adaptive survival mechanism of an endemic tree species in subtropical forest of eastern China, this study revealed that *C. oblongifolia* coordinated the physical protection (leaf thickening) and biochemical defense (secondary metabolite accumulation) through the gene regulatory network, thereby achieving the balance of resource utilization efficiency and stress tolerance under soil P limitation. The coupling adjustment of leaf morphology with the metabolism reflects a long-term evolutionary adaptation of perennial woody plants to P-limited ecosystems, thereby providing key scientific basis for ecological restoration, conservation planning, and genetic resource management. This is a complete analytical example of the environmental adaptation mechanism of a tree species with a small population. We expect to establish a replicable study model, ranging from soil nutrient limitation to molecular marker early warning, to provide precise protection for small populations like *C. oblongifolia*.

## Data Availability

The datasets presented in this study can be found in online repositories. The names of the repository/repositories and accession number(s) can be found in the article/**Supplementary Material**.
