# Supplementary material for: Multi-omics integration analysis of leaves reveals the adaptive survival mechanism of Carpinus oblongifolia, an endemic tree species in subtropical forests of eastern China
Source: Front Plant Sci. 2026 Sep 3;17:1920434. doi: 10.3389/fpls.2026.1920434 (PMC13582429; doi:10.3389/fpls.2026.1920434)
Supplement: Supplementary file 1 [file Table1.docx]

Supplementary Figures

**Supplementary Figure 1** The scale-free topology fit plot based on 22,481 genes for WGCNA. This plot was conducted in Omicsmart, a real-time interactive online platform for data analysis (http://www.omicsmart.com).

**Supplementary Figure 2** Histogram of 10 leaf traits of *Carpinus oblongifolia* among four habitats. LA, leaf area; LT, leaf thickness; PTT, palisade tissue thickness; STT, spongy tissue thickness; PSR, the ratio of PTT to STT; SLW, specific leaf weight; LDMC, leaf dry mass content; LN, leaf nitrogen concentration; LP, leaf phosphorus concentration; LNP, the ratio of LN to LP. Different letters indicated significant differences in traits among habitats at *P* < 0.05. Bars are mean values ± SD (n = 3).

**Supplementary Figure 3** RDA ranking comparison between four habitats and different subsets excluded a specific habitat. The analysis was conducted in Omicsmart based on six parameters of soil nutrients and three leaf traits of LT, PTT, and STT.

**Supplementary Figure 4** PCA and the number of differentially expressed genes across four habitats based on all transcriptome data.

**Supplementary Figure 5** The correlations between gene significance for LT, STT, SLW, PTT, and LDMC with Module Membership in blue module.

**Supplementary Figure 6** The correlations between gene significance for STT and LN with Module Membership in purple module.
